# Supplementary material for: High affinity nanobodies block SARS-CoV-2 spike receptor binding domain interaction with human angiotensin converting enzyme
Source: Sci Rep. 2020 Dec 22;10:22370. doi: 10.1038/s41598-020-79036-0 (PMC7755911; doi:10.1038/s41598-020-79036-0)
Supplement: Supplementary file 1 — Supplementary Information. [file 41598_2020_79036_MOESM1_ESM.docx]

**SUPPLEMENTARY MATERIAL**

**High Affinity Nanobodies Block SARS-CoV-2 Spike Receptor Binding Domain Interaction with Human Angiotensin Converting Enzyme**

Thomas J. Esparza^1,2^, Negin P. Martin^3,4^, George P. Anderson^5^, Ellen R. Goldman^5^, David L. Brody^1,6*^

^1^The National Institute of Neurological Disorders and Stroke Intramural Research Program, Laboratory of Functional and Molecular Imaging, Bethesda, MD, USA 20892

^2^Henry M. Jackson Foundation for the Advancement of Military Medicine, Bethesda, MD, USA 20892

^3^Viral Vector Core, National Institute of Environmental Health Sciences, NIH/DHHS, Research Triangle Park, NC, USA 27709.

^4^Neurobiology Laboratory, National Institute of Environmental Health Sciences, NIH/DHHS, Research Triangle Park, NC, USA 27709.

^5^US Naval Research Laboratory, Center for Biomolecular Science and Engineering, Washington, DC, USA 20375

^6^Department of Neurology, Uniformed Services University of the Health Sciences, Bethesda, MD, USA 20814

**Supplementary Fig. 1**


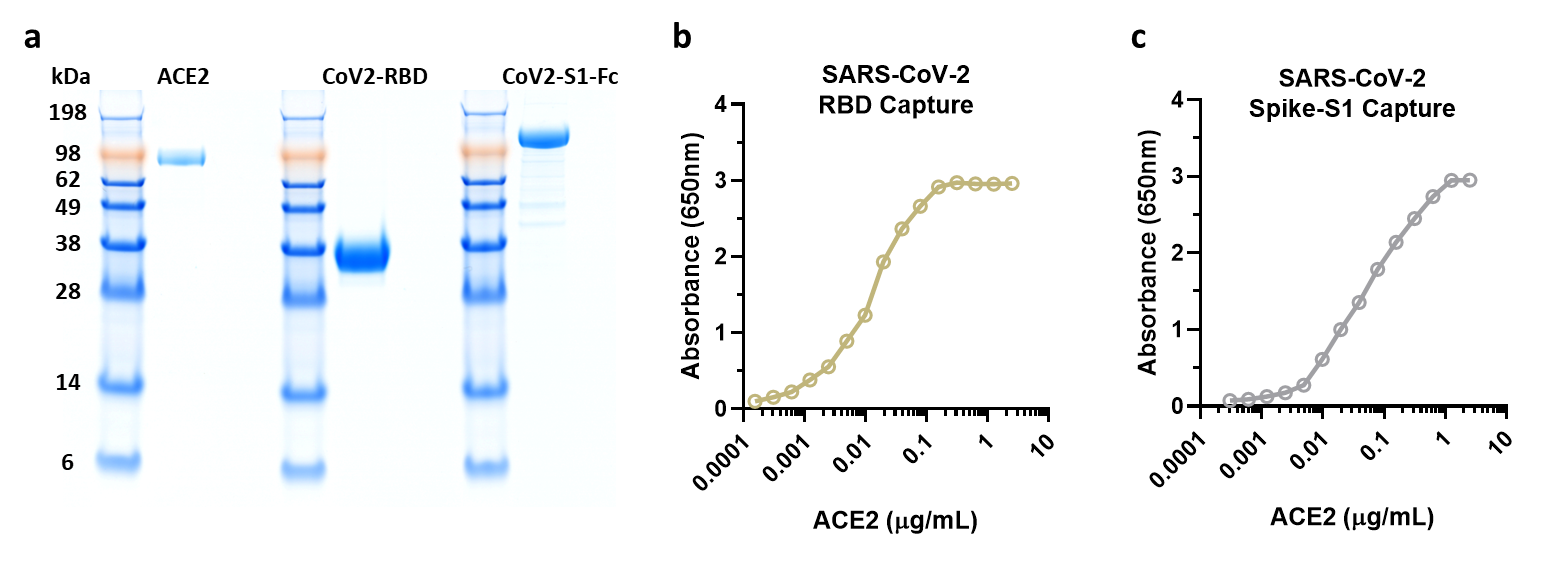


**Supplementary Fig. 1:** **Reagents required for characterization and validation include recombinant human ACE2, recombinant SARS-CoV-2 receptor binding domain (RBD), and recombinant SARS-CoV-2 Spike protein (S1)**. **a** The presence of prominent, single bands on a SDS-PAGE gel for each protein indicate purity and appropriate size. **b,c** Validation that recombinant SARS-CoV-2 RBD and SARS-CoV-2 Spike S1 bind with high affinity to recombinant human ACE2. This high affinity, saturable binding indicates that all 3 recombinant proteins are appropriately folded *in vitro*.

**Supplementary Fig. 2**


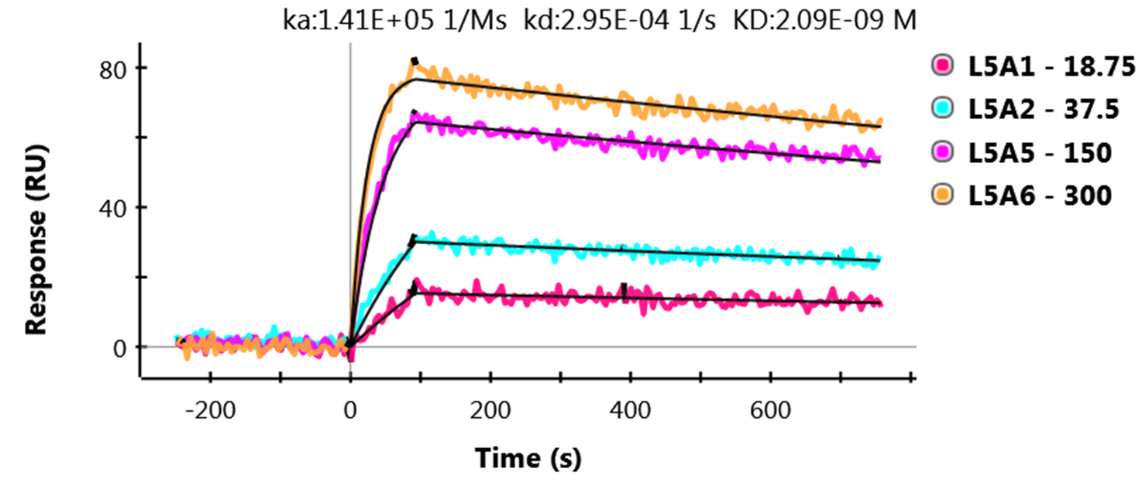


Supplementary Fig. 2: Surface plasmon resonance affinity measurement of NIH-CoVnb-112 binding RBD. Validation of the high affinity binding constant of NIH-CoVnb-112 was established by measurement of binding using an alternative affinity measure. SARS-CoV-2 RBD was immobilized onto the surface of a GLC chip using EDC/NHS chemistry followed by flow of NIH-CoVnb-112 across the chip surface to allow for measurement of binding kinetics. Raw sensor data is overlaid by software curve fits which allow for measurement of the association and dissociation constants using the Langmuir model. The calculated affinity binding constant (2.1nM) is in close agreement with the value measured with biolayer interferometry (4.9nM).

**Supplementary Fig. 3**


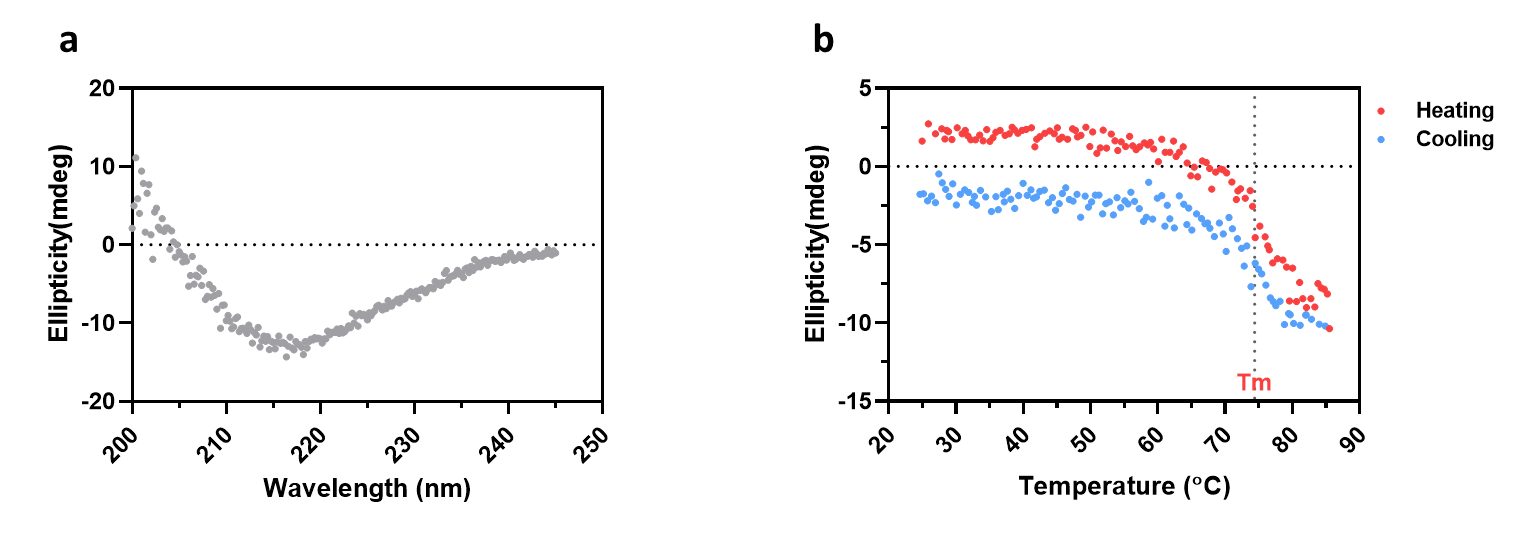


**Supplementary Fig. 3:** **Characterization of NIH-CoVnb-112 by circular dichroism.** **a** Representative CD curve for NIH-CoVnb-112. **b** Reversible folding was monitored using circular dichroism using a Jasco J-815 spectropolarimeter at 205nm during a heating-cooling cycle over 25°C to 85°C at a rate of 2.5 °C/min. The inflection point at 74.4°C indicates the melting temperature. Using the delta between the heating and cooling curves is used to calculate a 73% refolding rate for NIH-CoVnb-112 which is indicative of a highly stable structure.

**Supplementary Fig. 4**


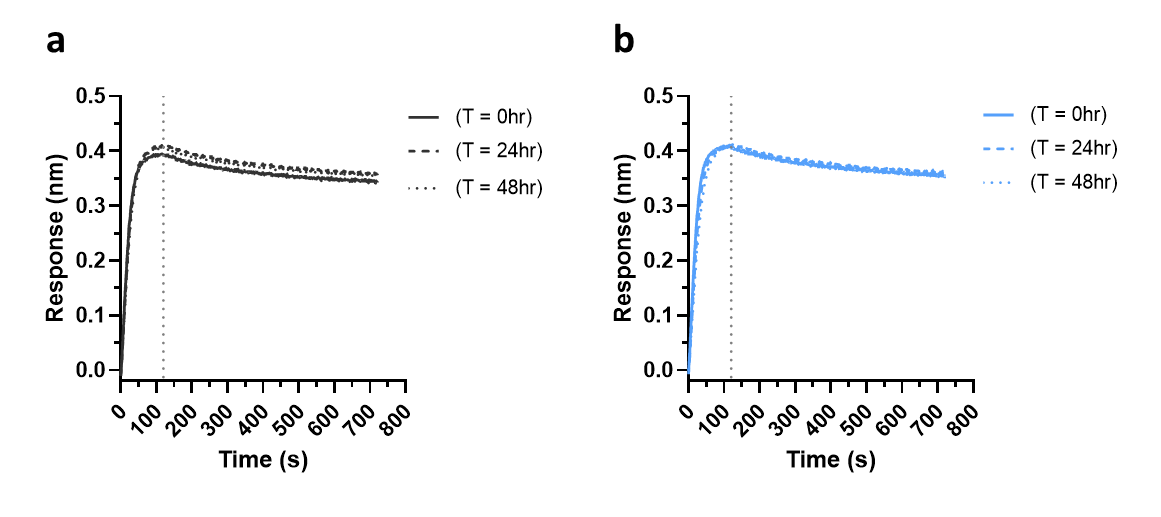


**Supplementary Fig. 4: NIH-CoVnb-112 is highly stable during controlled incubation with human plasma and albumin.** To determine the if NIH-CoVnb-112 is sensitive to plasma conditions a controlled incubation was performed at 37°C with mixing. Measurement of binding, following incubation for 24 or 48hrs, of NIH-CoVnb-112 in (**a)** pooled human plasma or (**b)** recombinant human albumin alone to mimic conditions which could lead to degradation and loss of function. NIH-CoVnb-112 was additionally spiked into pooled human plasma or recombinant human albumin immediately prior to measurement by biolayer interferometry.

**Supplementary Fig. 5**


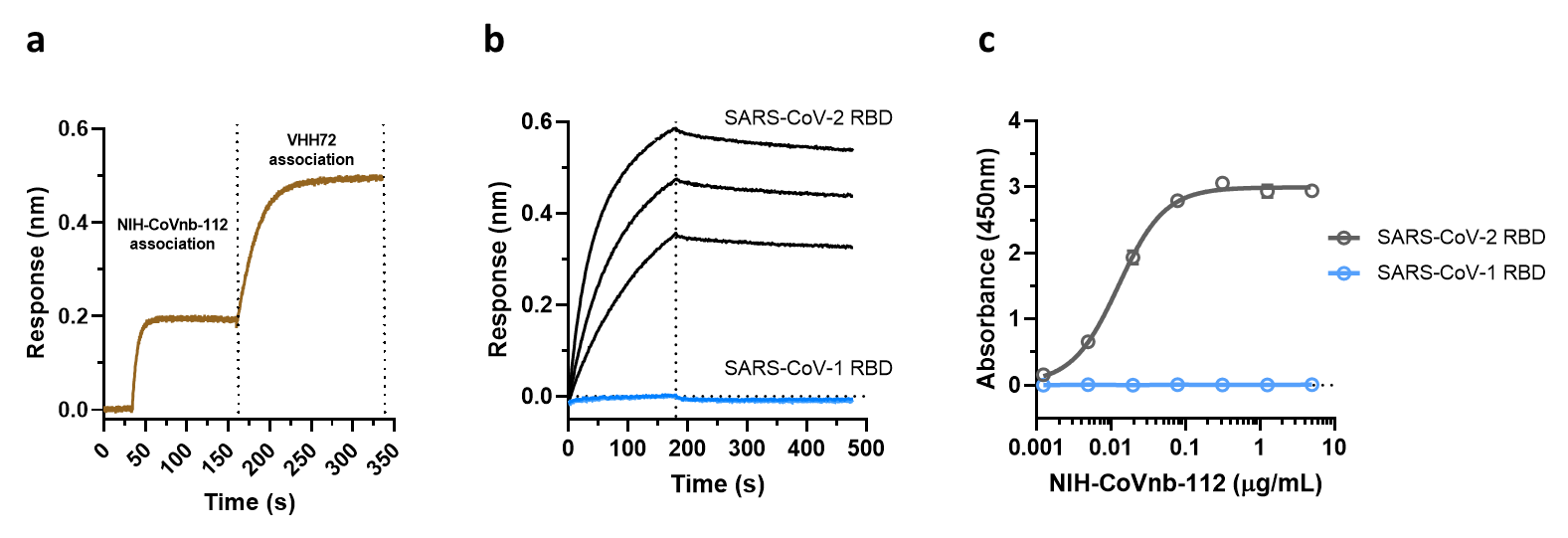


Supplementary Fig. 5: NIH-CoVnb-112 binds to SARS-CoV-2 RBD at a distinct epitope from that bound by VHH72 and does not bind to SARS-CoV-1 RBD. a To determine if NIH-CoVnb-112 and VHH72 have competing epitopes the following association Octet experiment was performed: Biotinylated SARS-CoV-2 RBD was bound to streptavidin BLI sensors in blocking buffer. Following baseline stability, the sensor was transferred into a well containing 500nM NIH-CoVnb-112 and allowed to associate. The sensor was then transferred to the adjacent well containing 500nM VHH72 and allowed to associate. If VHH72 had an overlapping epitope blocked by NIH-CoVnb-112 there would be minimal to no increase in response. Based on the observed increase in response, it can be inferred that the two nanobodies have non-competing epitopes on the SARS-CoV-2 RBD. b An Octet experiment was performed to determine if NIH-CoVnb-112 possesses permissive binding to SARS-CoV-1 RBD. NIH-CoVnb-112 was biotinylated using NHS-LC-biotin and then immobilized onto a streptavidin biosensor followed by association of SARS-CoV-1 RBD (*blue curves*) and SARS-CoV-2 (*black curves*) at 500, 250, and 125nM RBD. NIH-CoVnb-112 does not bind to SARS-CoV-1 RBD. c As an orthogonal confirmation of the Octet experiment, binding was measured by ELISA. SARS-CoV-1 RBD (*blue open circles*) and SARS-CoV-2 RBD (*black open circles*) were coated on to an ELISA plate at 10 micrograms/mL and incubated with a range of NIH-CoVnb-112 concentrations. An anti-alpaca secondary antibody was used for detection and confirms the lack of NIH-CoVnb-112 binding to SARS-CoV-1 RBD.

**Supplementary Fig. 6**


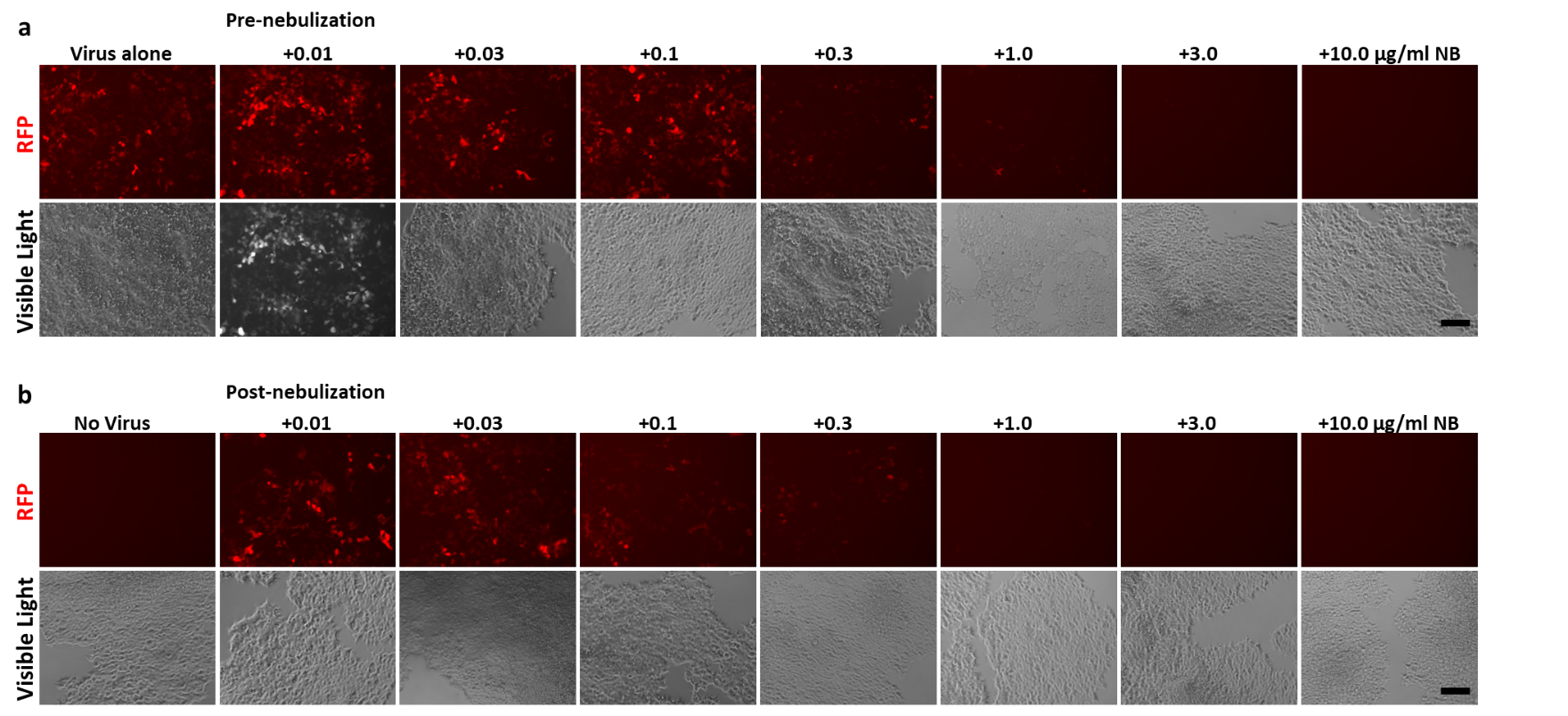


**Supplementary Fig. 6: Epifluorescence microscopy of inhibition of SARS-CoV-2 pseudotyped virus transduction by NIH-CoVnb-112.** Inhibition of a SARS-CoV-2 pseudovirus transduction was performed (MOI=0.5) on HEK293-ACE2 cells with a range of NIH-CoVnb-112 concentrations from nanobody before and after nebulization to mimic therapeutic inhaled administration. Prior to flow cytometry evaluation, fields in each well were imaged by brightfield and RFP epifluorescence for the (**a**) pre-nebulization and (**b**) post-nebulization samples. At and above concentrations of 1 microgram/mL NIH-CoVnb-112 there is a robust inhibition of fluorescence signal, while below 0.3 micrograms/mL there is a gradual increase to comparable levels to the virus alone control. This demonstrates the potent inhibition of the biologically active SARS-CoV-2 spike protein and the interaction with its receptor. (Scale bar = 100µm)

**Supplementary Fig. 7**


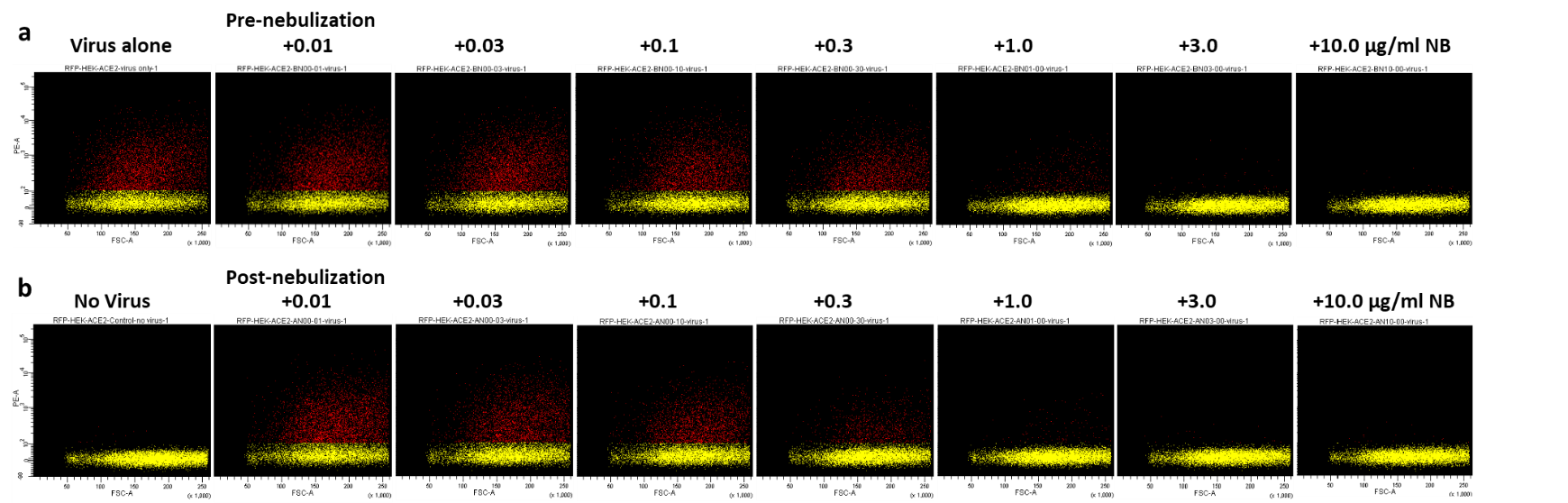


**Supplementary Fig. 7: Flow cytometry analysis of HEK293-ACE2 cells following inhibition of SARS-CoV-2 pseudotype virus by NIH-CoVnb-112.**  Following a 48hr transduction of SARS-CoV-2 pseudovirus in the presence of pre and post-nebulization NIH-CoVnb-112, at various concentrations, the cells were trypsinized and fixed prior to flow cytometry. A single cell population was gated, to exclude debris, and 10,000 events collected per sample on a BDFortessa in the PE-Cy5.5 channel in triplicate. The (**a**) pre-nebulization and (**b**) post-nebulization sample display a very similar distribution of RFP positive cells at each respective concentration of NIH-CoVnb-112. Yellow dots represent gated non-positive events; red dots represent gated positive events.
